# Energy Transfer Efficiency Based Nonlinear Ultrasonic Testing Technique for Debonding Defects of Aluminum Alloy Foam Sandwich Panels

**DOI:** 10.3390/s23063008

**Published:** 2023-03-10

**Authors:** Jun Tu, Nan Yao, Yi Ling, Xu Zhang, Xiaochun Song

**Affiliations:** 1 School of Mechanical Engineering, Hubei University of Technology, Wuhan 430068, China; juntu@hbut.edu.cn (J.T.);; 2 Hubei Key Laboratory of Modern Manufacturing Quality Engineering, Wuhan 430068, China

**Keywords:** nondestructive testing, nonlinear ultrasonic, aluminum alloy foam sandwich panels, thin plate, integral method

## Abstract

In order to improve the accuracy of detection results of debonding defects of aluminum alloy thin plate, the nonlinear ultrasonic technology is used to detect the simulated defect samples, aiming at problems such as near surface blind region caused by the interaction of incident wave, reflected wave and even second harmonic wave in a short time due to the small thickness of thin plates. An integral method based on energy transfer efficiency is proposed to calculate the nonlinear ultrasonic coefficient to characterize the debonding defects of thin plates. A series of simulated debonding defects of different sizes were made using aluminum alloy plates with four thicknesses of 1 mm, 2 mm, 3 mm and 10 mm. By comparing the traditional nonlinear coefficient with the integral nonlinear coefficient proposed in this paper, it is verified that both methods can quantitatively characterize the size of debonding defects. The nonlinear ultrasonic testing technology based on energy transfer efficiency has higher testing accuracy for thin plates.

## 1. Introduction

Aluminum alloy sheet structure has the characteristics of light weight and high strength, but its elastic modulus is low and the structural stability is weak. In order to improve this performance, the aluminum alloy sheet structure can be filled with polyurethane foam material, so that the thin-walled structure has higher strength and energy absorption performance. Usually, polyurethane foam [1] is formed by foaming process and bonded with aluminum alloy panel. However, in the process of bonding, due to immature technology, improper operation and other reasons, various defects will appear on the bonding surface such as poor bonding, pores and so on, which directly affect the safety and reliability of the whole structure. Therefore, it is necessary to study the nondestructive testing method for the bonding surface defects of aluminum alloy foam sandwich board.

There is a large density difference between aluminum alloy and polyurethane, which makes many methods (such as ray method [2], infrared thermal imaging method [3], ultrasonic phased method [4] and so on) for the detection of debonding defects unsatisfactory. At present, nonlinear ultrasonic detection technology is being used more and more in the detection of debonding defects. For cracks and holes in the structure, the conventional ultrasonic method has a high detection sensitivity [5], which mainly depends on the linear elastic theory of acoustics [6]. However, when the ultrasonic wave propagates in the material, the interface debonding defect will cause the nonlinear response of the ultrasonic wave, and then the time domain waveform of the received signal will be distorted, which is represented by the generation of higher-order harmonics in the frequency domain. Therefore, the ratio of the fundamental wave to the second harmonic wave (which means relative nonlinear coefficient β) is often used to characterize the microscopic defects in the measured material in nonlinear ultrasonic detection technology [7]. A lot of work has been performed on the nonlinear detection of debonding defects by scholars at home and abroad. Mondal et al. [8] proposed a nonlinear ultrasonic dislocation model earlier, and calculated the expressions of the nonlinear parameters. The influence of dislocation on harmonic amplitude is verified by using aluminum plates as experimental objects, which improved accuracy in characterizing material microscopic defects by nonlinear parameters. On that basis, Zhou et al. [9] proposed a nonlinear spring model of the bonding surface under vertical incidence of P-wave. The nonlinear term in the relation between the interface stress and the discontinuous displacement of the interface is used to accurately characterize the small changes of the bonding surface, and thus to characterize the debonding state of the material. Zhang et al. [10] established acoustic models and bilinear stiffness models of micro-bonding at different stages, which proved that the bonding property of composite insulation is related to the acoustic impedance of the interface and the nonlinear deformation of its constitutive relation. Additionally, the nonlinear distortion of high-power ultrasonic wave was used to diagnose the micro-bonding defects $\left(1\sim 20\;\mu m\right)$ at the early stage of the composite insulation interface [11].

However, the thickness of detected objects in all the above models is relatively large. Only the β coefficient of single reflection echo signal can be used for analysis. Additionally, the thickness of aluminum alloy in the foam sandwich board almost has a range of 1~5 mm. If the ultrasonic probe with a wafer diameter of 10 mm and a center frequency of 1 MHz is selected, the length of the near field region of ultrasonic detection [12] is 6.4 mm, which is equivalent to the thickness of the aluminum alloy, resulting in large amplitude fluctuation of the fundamental wave and the higher harmonic wave, and then using β coefficient to characterize nonlinear features will inevitably produce great errors. To solve this problem, in this paper a new characterization method is proposed to improve the detection accuracy of debonding defects in aluminum alloy foam sandwich panels. The main idea is to treat the generation of the second harmonic as the transmission of energy from the fundamental wave to the frequency segment of the second harmonic wave, and the energy transfer efficiency γ is used to characterize the degree of nonlinear debonding.

## 2. Nonlinear Ultrasonic Debonding Detection Theory of Thin Plate

### 2.1. Traditional Nonlinear Ultrasound

According to the nonlinear acoustic theory in solids [13], the relationship between stress σ and strain ε can be obtained as

(1) $$\sigma =E\left(1+\frac{1}{2}\beta \varepsilon^{2}+\frac{1}{3}\delta \varepsilon^{3}\cdots \right)$$

where E is the elastic modulus, and β and δ are the second- and third-order nonlinear parameters related to the material properties.

The nonlinear equation of sound wave propagation in the medium has different manifestations in different coordinate systems. It is simple and effective to use the plane wave equation in Lagrange coordinates [14]. Its one-dimensional wave equation is

(2) $$\rho \frac{\partial^{2}u}{\partial t^{2}}=\frac{\partial \sigma }{\partial x}$$

where ρ is the material density, and $u\left(x,t\right)$ and $\sigma \left(x,t\right)$ are the displacement and stress of the vibrating particle in the x direction.

Material deformation is often considered to be very small in traditional nonlinear ultrasonic detection [15]. Then, the strain $\varepsilon \left(x,t\right)$ in the direction of x can be simply expressed as

(3) $$\varepsilon =\frac{\partial u}{\partial x}$$

According to the characteristics of the ultrasonic sound field, there is a near surface blind region due to wave interference. Therefore, a non-uniform distribution of sound pressure is generated around the wave source. The testing object in this paper is aluminum alloy sheet, so the effect of the near-surface blind region cannot be ignored. Therefore, Equation (3) is further modified as

(4) $$\varepsilon =\frac{\partial u}{\partial x}-\frac{\partial \left(u+h\right)}{\partial x}+\frac{\partial \left(u+2h\right)}{\partial x}-\cdots$$

The first term on the right side of Equation (4) is the fundamental strain, the second term is the strain of the second harmonic wave and the third term is the strain of the fundamental wave echo [16].

By combining Equations (1), (2) and (4) and ignoring higher terms above the third order, the displacement equation of the particle can be obtained as follows:

(5) $$\frac{\partial^{2}u}{\partial t^{2}}=c^{2}\left(1+\beta \left(\frac{\partial u}{\partial x}-\frac{\partial \left(u+h\right)}{\partial x}+\frac{\partial \left(u+2h\right)}{\partial x}\right)+\delta {\left(\frac{\partial u}{\partial x}-\frac{\partial \left(u+h\right)}{\partial x}+\frac{\partial \left(u+2h\right)}{\partial x}\right)}^{2}\right)\left(\frac{\partial^{2}u}{\partial x^{2}}-\frac{\partial^{2}\left(u+h\right)}{\partial x^{2}}+\frac{\partial^{2}\left(u+2h\right)}{\partial x^{2}}\right)$$

where $c=\sqrt{E∕\rho }$ is the wave speed.

Generally, there is no general analytic solution for the motion equation of a particle, so the perturbation method can be used to obtain an approximate solution [17]. $u\left(x,t\right)$ is expanded by a power series of x as

(6) $$u\left(x,t\right)=u_{0}\left(x,t\right)+xu_{1}\left(x,t\right)+x^{2}u_{2}\left(x,t\right)$$

However, the solution of Equation (5) by using the perturbation method has many limitations. The polynomial expansion has no solution, and this method can only be solved in the relevant region [18]. Therefore, a new method is needed to solve the problem of nonlinear ultrasonic plate debonding.

### 2.2. Energy Transfer Integral Method

Because the second harmonic in the traditional nonlinear ultrasonic detection is weak, it needs to be artificially amplified, so it is rare to associate the fundamental wave with the second harmonic by energy transfer, and also the attenuation caused by sound waves propagating through isotropic solids is often ignored [19]. However, the testing object in this research is thin plate, the propagation distance is very close and the energy attenuation is very small, so the magnitude of the second harmonic is very significant. In this case, the generation of higher harmonics can be regarded as the transfer of acoustic energy from low frequency to high frequency. The transfer efficiency of energy can be used to characterize the nonlinear parameter of the thin plate.

First, the concept of energy spectrum is introduced, and the square of the amplitude spectrum of the frequency domain signal obtained by the Fourier transform is called the energy spectrum, which represents the amount of energy per unit frequency band, denoted by E. In order to further study the law of energy variation with frequency, the concept of energy spectral density is introduced. There are two methods to solve the energy spectral density [20]: (1) the autocorrelation function of the signal is solved first, and then the Fourier transform is performed; (2) take the Fourier transform first, and then square its amplitude. The second method is adopted in this paper. The RAM-5000SNAP nonlinear high-energy acoustic detection system is used in this experiment. The excitation signal is the radio frequency pulse signal, which can be considered as a square integrable continuous signal $f\left(t\right),$ and the Fourier transform is performed. The square of its amplitude is the energy spectral density $\varphi \left(\omega \right)$

(7) $$\varphi \left(\omega \right)={\left|\frac{1}{2\pi }\int_{-\infty }^{\infty }f\left(t\right)\exp\left(-\omega t\right)dt\right|}^{2}=\frac{{\left|F\left(\omega \right)\right|}^{2}}{4\pi^{2}}$$

According to theorem Parseval [21], the total energy of ultrasonic wave is the integral of the energy spectral density function in the whole frequency interval

(8) $$E=\frac{1}{2\pi }\int_{-\infty }^{\infty }{\left|F\left(\omega \right)\right|}^{2}d\omega =2\pi \int_{-\infty }^{\infty }\varphi \left(\omega \right)d\omega =2\pi \int_{-\infty }^{\infty }\varphi \left(f\right)df$$

From Equation (8), the energy of the nonlinear ultrasonic signal in the fundamental wave frequency band and the second harmonic frequency band can be expressed as the integral in the interval $\left[f_{1},f_{2}\right]$ and $\left[f_{3},f_{4}\right]$, respectively, as follows:

(9) $$E_{1}=2\pi \int_{f_{1}}^{f_{2}}\varphi \left(f\right)df$$

(10) $$E_{2}=2\pi \int_{f_{3}}^{f_{4}}\varphi \left(f\right)df$$

Finally, the nonlinear coefficient γ representing the discontinuous debonding degree of materials can be expressed as follows:

(11) $$\gamma =\frac{E_{2}}{E_{1}}=\frac{\int_{f_{3}}^{f_{4}}\varphi \left(f\right)df}{\int_{f_{1}}^{f_{2}}\varphi \left(f\right)df}$$

## 3. Experimental Study

### 3.1. Testing Platform

**Figure 1:**
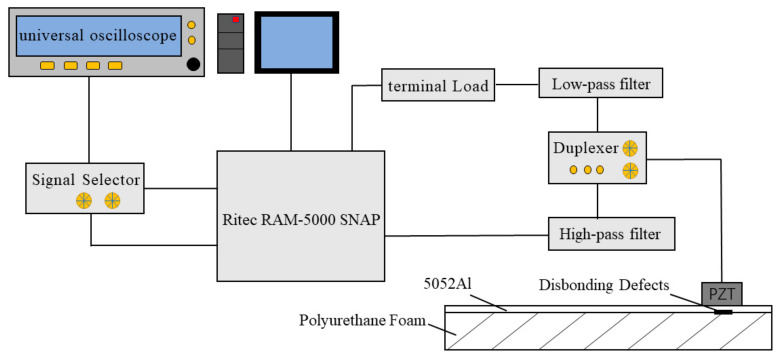
Frame diagram of the experimental platform.

The experimental testing platform was built as shown in Figure 1. It includes an RF pulse transmitter and receiver (Model: RAM-5000), duplexer (Model: RDX-6), signal selector (Model: RS-5-G2), terminal load (Model: RT-50), filter bank (Model: RLP/RHP), 0.5 Mhz piezoelectric probe (Model: V101-RB/1), 1 Mhz piezoelectric probe (Model: V103-RB/0.5), 2.25 Mhz piezoelectric probe (Model: V106-RB/0.5), 5 Mhz piezoelectric probe (Model: V109-RB/0.5) and a universal oscilloscope (Model: GDS-2102E). These probes are from Olympus and their specifications are shown in Table 1 below. Each probe is separately connected to the instrument and the corresponding filter bank is used to obtain better testing results.

**Figure 2:**
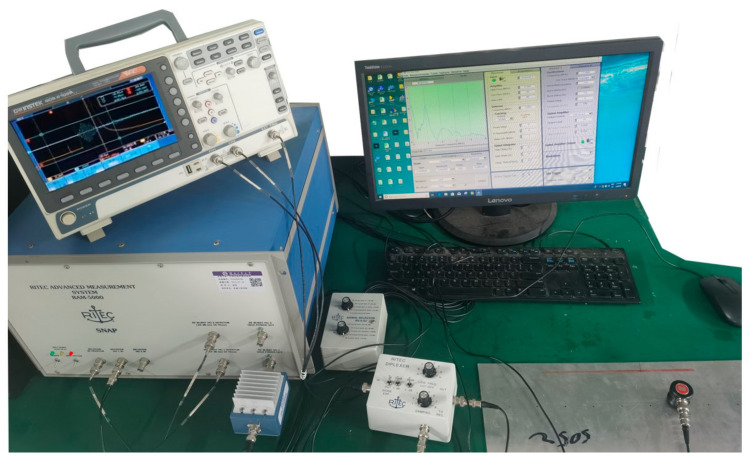
The schematic diagram of experimental operation.

The schematic diagram of experimental operation is shown in Figure 2. After the instrument is connected, measurement is carried out according to the marked points. Observe the oscilloscope and frame the first echo with a hanning window. Set the RETIC interface parameters as follows: center frequency 1 MHz, pulse number 5, gain 50 dB. Click start to obtain the frequency map of the point on the computer.

### 3.2. Sample Preparation

In order to validate the effectiveness of the proposed method in thin plate detection, four kinds of aluminum plates with thicknesses of 1 mm, 2 mm, 3 mm and 10 mm were selected to make test samples. An amount of 3 M polyurethane foam tape is used as the core layer material. Because the ultrasonic attenuation of polyurethane foam is very great and the acoustic impedance of polyurethane foam is very different from that of aluminum plate, the ultrasonic energy transmitted to the core layer of foam is very small, and the reflected energy is almost zero. Therefore, only polyurethane tape with a thickness of 3 mm was used. 

In general, three methods are often used to simulate the debonding defects of plates. (1) Punching method: After punching the plate, another two plates are fitted together. This method is usually suitable for the three-layer plate structure. (2) Trenching method: The surface to be measured is grooved and then fitted on one side. This method is usually suitable for the destructive double-layer plate structure. (3) Filling method: This method is used to fill the void with filler to form a debonding defect, which is often suitable for non-destructible structures. Given the widespread use of aluminum sheet in aircraft skin, it is not suitable for destructive methods and is not suitable for liquid fillings. Therefore, the third method is adopted in this paper to fill the paper into the void to form the debonding defect.

**Figure 3:**
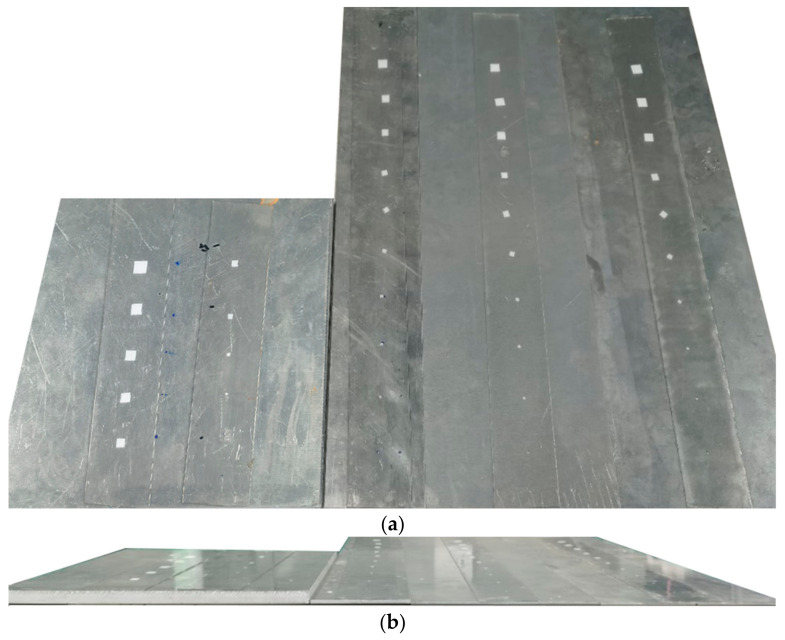
Sample physical drawing (**a**) Top view; (**b**) Side view.

In order to design standard debonding defects of different sizes, a 0.1 mm thick paper was placed between the aluminum plate and the tape to simulate the debonding defect, and the size of the paper was controlled to form defects of different sizes. The specific sizes of samples and defects are shown in Table 2. All defects were arranged with an interval of 40 mm, as shown in Figure 3. After the polyurethane foam was attached to the aluminum plate, the sample was pressed evenly with heavy objects for two hours to achieve a completely tight condition. The relationship between the standard defect size and the nonlinear parameters is established with the manual defect samples. Among them, the 10 mm thick sample is used to verify the influence brought by the near field region. It is also used to test the accuracy of the nonlinear ultrasonic testing technology proposed in this paper based on energy transfer efficiency.

The RAM-5000 excitation system was used to transmit 1 MHz high-voltage pulse through the load terminal to the piezoelectric ultrasonic probe, which was place on the defect testing point, and the reflecting wave single was displayed on the oscilloscope. Then, a complete echo waveform was tuned by setting the hanning window, and the RAM-5000 started to draw the frequency map. Every point with a defect was tested with the same procedure, and the peak amplitudes of the fundamental frequency and second harmonic frequency were recorded. For comparison, the traditional nonlinear coefficient $\beta =A_{2}/A_{1}^{2}$ was calculated first for each data. Then, the new nonlinear coefficient through the energy transfer integral method proposed in this paper was calculated according to Equation (11).

### 3.3. Nonlinear Ultrasonic Characteristics in Thin Plate

**Figure 4:**
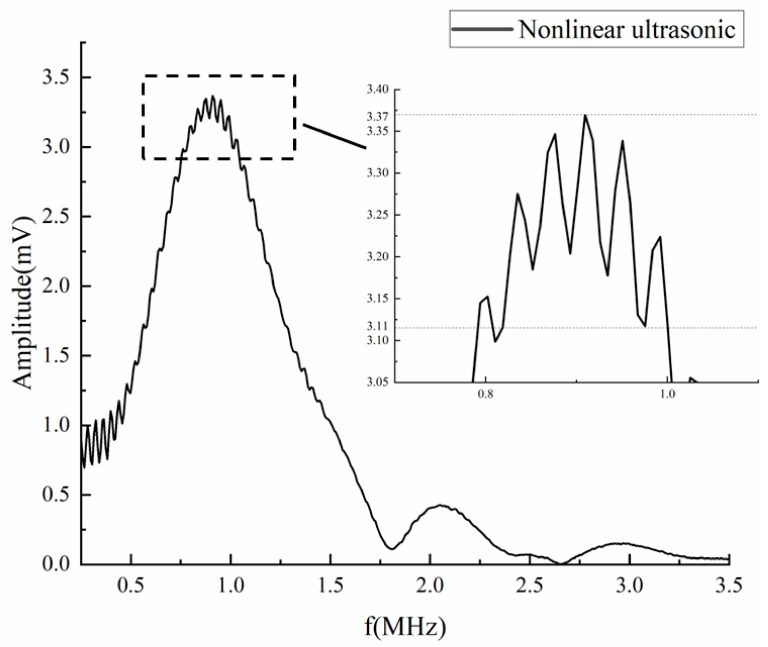
Frequency domain waveform and local amplification.

Firstly, a 1 MHz piezoelectric probe was used to carry out nonlinear ultrasonic detection experiment on the defect part of sample 1#. The frequency amplitude including fundamental wave and second harmonic wave can be obtained as shown in Figure 4. During the whole test process, the probe position and excitation parameters were kept unchanged, and the test was repeated at the same test point. Through local amplification around 1 MHz in Figure 4, it is found that the peak value of the fundamental wave fluctuates between 3.11 mV and 3.37 mV, and the difference of the maximum value can reach 0.26 mV. Similarly, the peak value of the second harmonic fluctuates between 0.37 mV and 0.45 mV, and the difference of the maximum values can reach 0.08 mV. This indicates that when the plate thickness is in the near field, the amplitude of ultrasonic detection signal is easily fluctuated. If the traditional nonlinear ultrasonic coefficient β is used to characterize the size of the debonding defect, the accuracy will be greatly affected.

**Figure 5:**
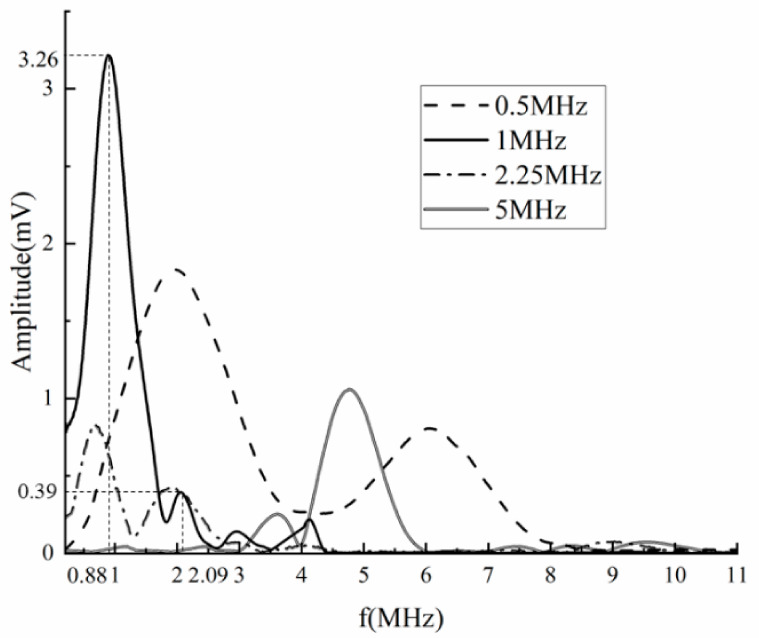
Spectrum of fundamental wave and second harmonic wave in frequency domain obtained by different center frequency probes.

In order to further explore the nonlinear ultrasonic characteristics in thin plate, probes with center frequencies of 0.5 MHz, 1 MHz, 2.25 MHz and 5 MHz, respectively, were used to test on the same test point of sample 1#, and the results are shown in Figure 5. It can be found that the peak position of the fundamental wave and the second harmonic wave will change with the probe center frequency. For the experiments with 1 MHz Probe, the fundamental wave crest is 3.26 mV, and the corresponding frequency is 0.88 MHz. The second harmonic peak is 0.39 mV, and the corresponding frequency is 2.09 MHz. Similarly, when other probes are used in the experiments, the same phenomenon will also appear, the frequency corresponding to the peak value is inconsistent with the center frequency of the probe. It is verified that the interference phenomenon in the near surface blind area will cause the frequency of the received signal to shift. Therefore, it is not accurate to use the amplitude of the center frequency to calculate the nonlinear ultrasonic coefficient, which will lead to a great error in the detection of debonding defects.

### 3.4. Quantitative Test of Debonding Defects

**Figure 6:**
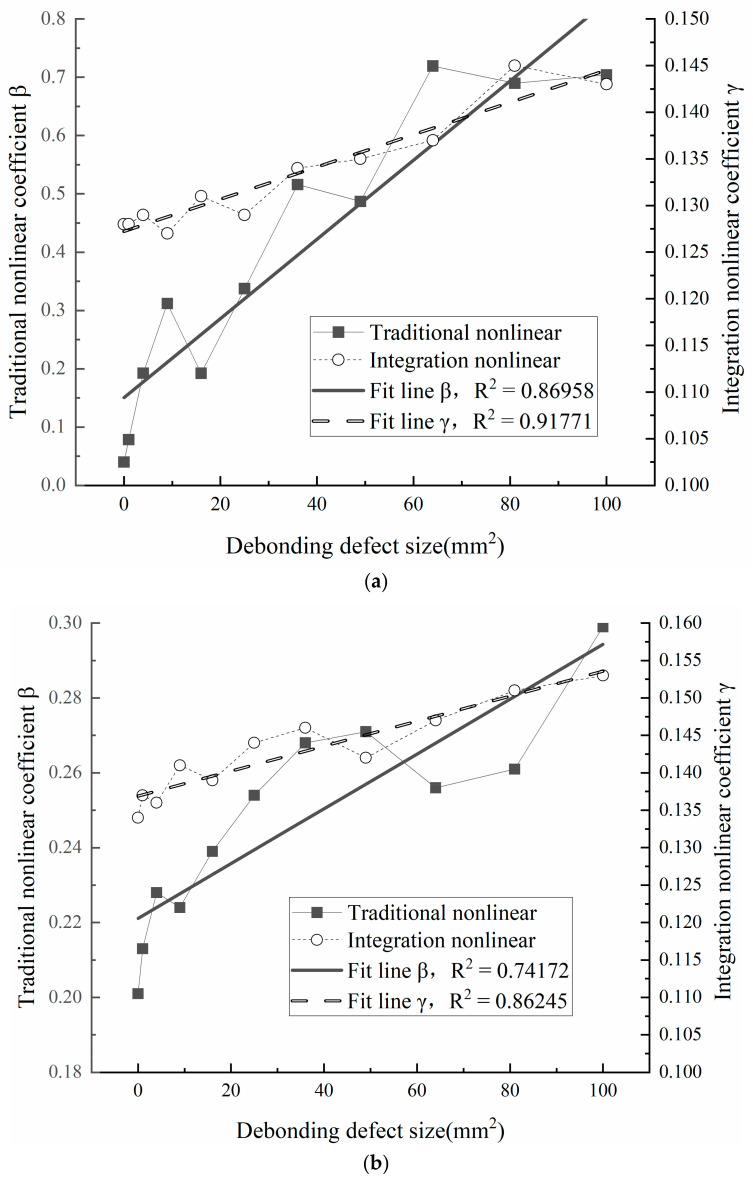

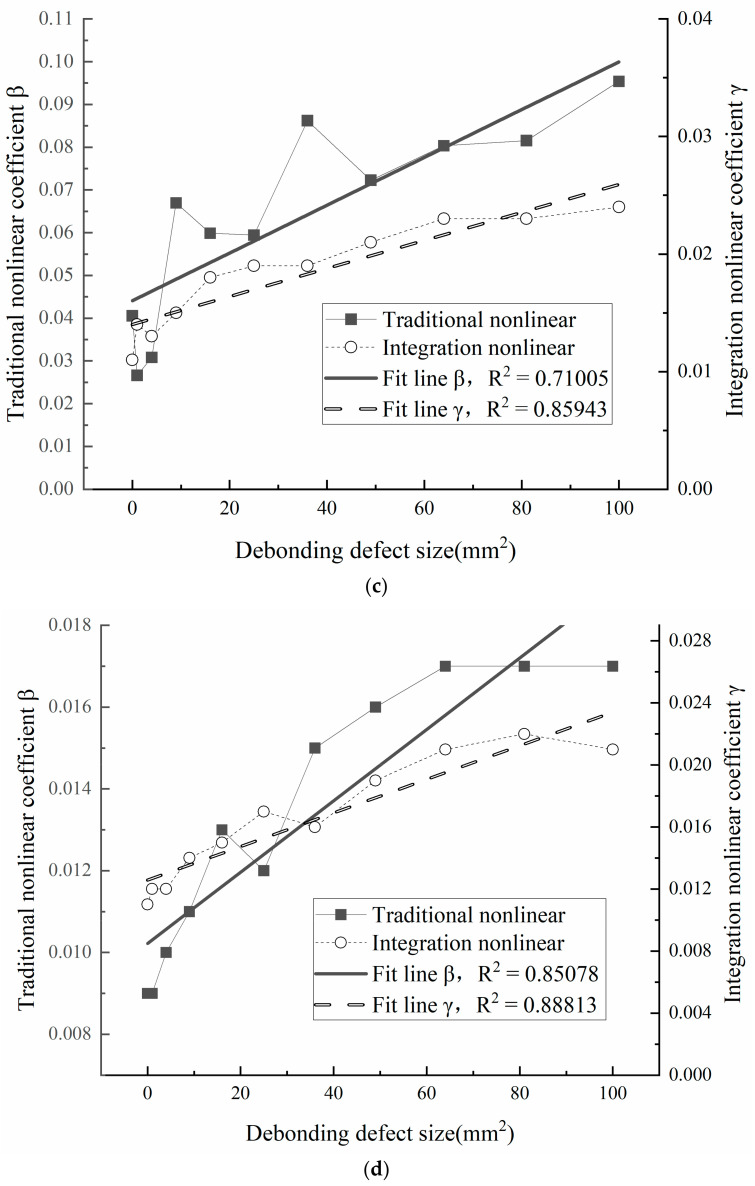
Fitting curves of the relation between defect size and relative nonlinear coefficient of four samples: (**a**) 1#sample, (**b**) 2#sample, (**c**) 3#sample, (**d**) 4#sample.

In order to verify the validity of the nonlinear coefficient calculation method proposed in this paper, the nonlinear ultrasonic tests were carried out on 44 test points of 4 aluminum alloy sheets from 1# to 4#. The corresponding amplitude of fundamental wave $A_{1}$ and second harmonic wave $A_{2}$ are read, which are used to calculate the traditional nonlinear coefficient β. In addition, the nonlinear coefficient γ represented by energy transfer efficiency was also calculated according to Equation (11). The comparison results of the two calculation methods are shown in Figure 6. It can be found that for the same sample, with the increase in defect size, the relative amplitude of the fundamental wave will decrease, the relative amplitude of the second harmonic wave will increase, and the nonlinear coefficients β and γ will increase.

Furthermore, linear fitting was carried out for the nonlinear coefficients corresponding to the debonding defects of different sizes in Figure 6. The determinable coefficient $R^{2}$ in the unitary linear fitting equation was used to measure the goodness-of-fit. It is not difficult to discover in Figure 6a–c that within the near surface blind region, the thinner the specimen is, the closer the $R^{2}$ of each test point is to 1, and the greater the $R^{2}$ calculated by the integral method. That is to say, the integral method is more accurate in characterizing debonding defects. With the increase in plate thickness, the $R^{2}$ of both the traditional nonlinear method and the integral method decreases, that is, the accuracy of characterizing the debonding defects is decreasing. 

Figure 6d shows the test results of sample 4#. It is obvious that $R^{2}$ of the nonlinear coefficient of the integral method is still closer to 1, and the gap between the integral method and the traditional method decreases with the increase in plate thickness. Obviously, the accuracy of the integral method is higher when the plate thickness is small, and the accuracy of the traditional method will increase as the plate thickness exceeds the near field length. It is reasonable to speculate that the accuracy of the two methods will be closer and closer when the plate thickness is larger. Therefore, the energy integral method proposed in this paper can be used as an effective supplement to the nonlinear ultrasonic detection of plate debonding defects, and further improve the detection accuracy of nonlinear ultrasonic debonding defects.

## 4. Conclusions

Nonlinear ultrasonic technology was used to detect the debonding defects. In order to improve the application of this technique in the testing of thin plate, the energy spectral density is introduced and the integral method of nonlinear ultrasonic detection based on energy transfer efficiency is proposed. Through a lot of experiments, conclusions are drawn as follows:

1. The nonlinear coefficient has a monotone correspondence with the size of the debonding defect. With the increase in the size of the debonding defect, the nonlinear characteristics will be significantly enhanced, and the relative nonlinear coefficient will also increase.
2. Plate thickness will greatly affect the nonlinear coefficient in the detection of debonding defects. When the plate thickness is less than the near-surface blind region of ultrasonic probe, the generation of second harmonic wave will be affected by the fundamental wave echo, thus affecting the traditional nonlinear coefficient. The energy transfer efficiency integral method presented in this paper to characterize the nonlinear coefficients can effectively improve the detection accuracy.
3. Compared with the traditional nonlinear coefficient method, the integral method can be used as an effective supplement for the detection of debonding defects in the near surface blind region.

## Figures and Tables

**Table 1:** Probe detail parameter.

Frequency (Mhz)	Nominal Element Size (in/mm)	Transducer Type
0.5	1/25	VIDEOSCAN V101-RB
1	0.5/12.7	VIDEOSCAN V103-RB
2.25	0.5/12.7	VIDEOSCAN V106-RB
5	0.5/12.7	VIDEOSCAN V109-RB

**Table 2:** Technical specifications of simulated debonding defects test samples.

Sample Number	Sample Size (mm)	Debonding Defects Size $\left({\mathrm{mm}}^{2}\right)$
1#	1000 × 150 × 1	0, 1× 1, 2×2, 3× 3, 4× 4, 5×5, 6× 6, 7× 7, 8×8, 9× 9, 10×10
2#	1000 × 150 × 2	0, 1× 1, 2×2, 3× 3, 4× 4, 5×5, 6× 6, 7× 7, 8×8, 9× 9, 10×10
3#	1000 × 150 × 3	0, 1× 1, 2×2, 3× 3, 4× 4, 5×5, 6× 6, 7× 7, 8×8, 9× 9, 10×10
4#	250 × 250 × 10	0, 1× 1, 2×2, 3× 3, 4× 4, 5×5, 6× 6, 7× 7, 8×8, 9× 9, 10×10

## Data Availability

Not applicable.
